# Comparison of Electrochemical Methods for the Evaluation of Cast AZ91 Magnesium Alloy

**DOI:** 10.3390/ma9110925

**Published:** 2016-11-15

**Authors:** Jakub Tkacz, Jozef Minda, Stanislava Fintová, Jaromír Wasserbauer

**Affiliations:** 1 Materials Research Centre, Faculty of Chemistry, Brno University of Technology, Brno 612 00, Czech Republic; xcminda@fch.vut.cz (J.M.); fintova@ipm.cz (S.F.); wasserbauer@fch.vut.cz (J.W.); 2 Institute of Physics of Materials, Academy of Sciences of the Czech Republic v. v. i., Žižkova 22, Brno 616 62, Czech Republic

**Keywords:** AZ91 magnesium alloy, cathodic polarization curve, anodic polarization curve, linear polarization curve

## Abstract

Linear polarization is a potentiodynamic method used for electrochemical characterization of materials. Obtained values of corrosion potential and corrosion current density offer information about material behavior in corrosion environments from the thermodynamic and kinetic points of view, respectively. The present study offers a comparison of applications of the linear polarization method (from −100 mV to +200 mV vs. E_OCP_), the cathodic polarization of the specimen (−100 mV vs. E_OCP_), and the anodic polarization of the specimen (+100 mV vs. E_OCP_), and a discussion of the differences in the obtained values of the electrochemical characteristics of cast AZ91 magnesium alloy. The corrosion current density obtained by cathodic polarization was similar to the corrosion current density obtained by linear polarization, while a lower value was obtained by anodic polarization. Signs of corrosion attack were observed only in the case of linear polarization including cathodic and anodic polarization of the specimen.

## 1. Introduction

Magnesium alloys are used in automotive and aerospace industries, as well as in computers, cell phones, sports equipment, and for many other applications. The wide range of applications of magnesium alloys is due to the good strength to weight ratio, good mechanical properties, and low density [1,2,3,4,5,6,7,8]. The possibility to tailor the material properties by changing the chemical composition and adequate mechanical and chemical treatment is also important. Many magnesium alloys are also promising to be used as biodegradable materials for medical applications [9,10,11,12,13,14,15,16,17,18,19,20,21,22,23,24,25,26,27,28,29,30,31,32,33]. Magnesium is nontoxic and biocompatible [15,19,21,34,35]. Magnesium ions also have very important biological functions; for example, magnesium takes part in bone and mineral homeostasis [29,36,37,38], promoting DNA replication and transcription [29,37,39], and regulation of opening and closing of ion channels [29,37,38,40,41,42]. When magnesium implants are used for bone support, they also supports the bone tissue to grow [29,43].

The high reactivity of magnesium and its alloys in a corrosion environment, especially in Cl^−^ solutions, limits their application in the engineering practice and also for medical applications. If the magnesium-based implant corrodes too fast (corrosion rate is too high), new bone tissue cannot replace already-corroded implant parts. The supporting effect of the implant and the advantage of similar strength and modulus of the magnesium-based materials to human bone is lost [9,29,43,44,45,46,47,48]. The corrosion of magnesium alloys is also accompanied by hydrogen evolution and intense alkalization of the surrounding tissues, which is a problem not only for orthopedic implants but also for cardiovascular stents [9,49].

Corrosion resistance of magnesium alloys and their corrosion rate can be basically affected by the chemical composition of the alloy (especially by controlling the impurities, such as Fe and Cu), mechanical or thermal treatment (with the aim to reach a homogeneous microstructure or to obtain a microstructure that controls the corrosion attack), or by surface treatment, especially coating (conversion, ceramic, etc.) [9,29,50,51,52,53,54,55,56,57,58,59,60,61,62].

AZ group magnesium alloy enhanced corrosion properties are reached due to the content of Al in the solid solution and is even improved by the addition of Zn. Content of Al also influences the amount of Mg_17_Al_12_ intermetallic phase particles, which affects the corrosion processes. The influence of Mg_17_Al_12_ particles on the alloy corrosion properties depends on their amount, morphology, and distribution in the alloy structure. In the case of cast alloys, the phase present in the eutectic form act as a barrier against corrosion propagation through the solid solution (lower corrosion resistance comparing to the intermetallic phase) and retard the corrosion evolution. However, the present intermetallic particles can also act as the cathode in microgalvanic cells and accelerate the corrosion process. This was observed in the case of material after mechanical treatment, where the Mg_17_Al_12_ intermetallic phase was present in the structure in the form of small localized particles. On the other hand, in the case of a homogenous distribution of very fine Mg_17_Al_12_ particles in magnesium alloy structure, a positive influence on the corrosion behavior can be reached. Very fine structure of AZ magnesium alloys reached after severe plastic deformation treatment had positive influence on the alloy corrosion properties due to the uniform corrosion of the alloy surface and protection of the material against the contact with the corrosion environment via the created corrosion products covering the whole surface. In addition to the present intermetallic phases, the grain boundaries, which are normally cathodic compared with the body of the grains, also influence the corrosion attack evolution [63,64,65,66,67,68].

Corrosion behavior of magnesium alloys can be analyzed by several methods. The methods can be basically divided into: (i) short-term methods (electrochemical methods, such as potentiodynamic polarization or electrochemical impedance spectroscopy); and (ii) longer term methods (such as mass loss or hydrogen gas collection). Both types of the methods have their specifics and the obtained data have to be interpreted carefully. Data obtained by the short-term methods may not be indicative of long-term corrosion, while the material reactions in the corrosive environment change with the exposure time (a protective layer is created, broken, recreated, etc.). The data obtained during the long-term experiments have to take into account the experiment conditions; especially the duration of the experiment has to be set precisely. Depending on the used method the determined corrosion rates of magnesium and its alloys can differ. Corrosion rates estimated based on the results obtained by electrochemical methods are usually lower when compared to the other methods, which is a result of changing the corrosion rate of the material in time due to the changing reactivity of magnesium due to the changing pH on the surface [69].

The advantage of electrochemical methods is in the continuous monitoring of the corrosion process during the relatively short exposure time. However, electrochemical methods can only follow corrosive process due to electrochemical dissolution. When other chemical reactions participate in the corrosion process, corrosion rates determined based on electrochemical method results might be much lower compared to the values determined by weight loss measurements, volume of hydrogen gas, or amount of corrosion products in the solution. The differences in the determined values depend on the corrosion behavior of the exact material and also change in the case of different alloys due to the chemical composition. Pardo et al. [68] showed good agreement in the corrosion rates estimated by electrochemical methods and mass loss measurements for AZ80 and AZ91D, however, different values were determined for pure Mg and AZ31 (lower values were estimated by electrochemical methods).

Potentiodynamic measurements performed via linear polarization are usually used to obtain polarization Tafel curve,s from which the corrosion potential (E_corr_) and the corrosion current density (i_corr_) can be determined. Potentiodynamic polarization shows the information about the corrosion process kineticss and it is the only method that can reveal the relative anodic and cathodic contributions. The method is destructive in nature and cannot serve for prediction of the long-term corrosion rates of the material [69].

**Figure 1:**
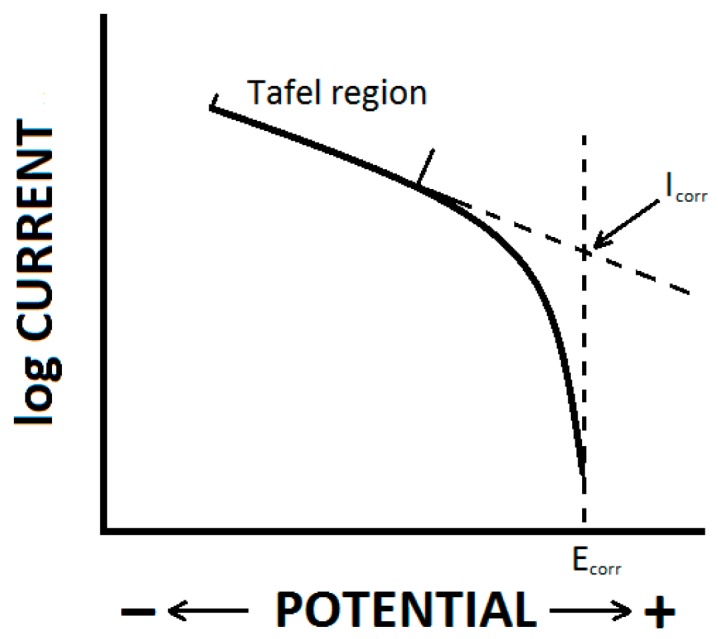
Tafel extrapolation of the cathodic part of the polarization curve.

The evaluation of the obtained polarization curves used in Tafel analysis are based on the extrapolation of the linear parts of the obtained curves. Tafel regions used for the polarization curve evaluation start at approximately 50 mV (usually up to 100 mV) from the corrosion potential (E_corr_), and the open circuit potential (E_OCP_) in the steady state, in the cathodic, as well as the anodic, branches of the polarization curve. The Tafel region is characterized by linear dependence of i_corr_ on E_corr_. Figure 1 shows Tafel extrapolation of the Tafel region (linear part of the anodic branch of the polarization curve) which is used to evaluate potentiodynamic characteristics to obtain the corrosion current I_corr_ from which the corrosion current density i_corr_ can be calculated (i_corr_ = I_corr_/specimen area immersed in the corrosion environment) [70,71]. 

For many metals and alloys exhibiting active-passive behavior, the anodic part of the polarization curve should not be used for the evaluation of material behavior because of the absence of the linear Tafel region. There are some other limitations for the Tafel analysis of the polarization curve caused by the mechanism of the corrosion process. The cathodic branch of the polarization curve cannot be used for Tafel analysis when the corrosion is under diffusion control; decreasing potential enters into the diffusion control region or the nature of the interface changes with the changing potential [70,71].

For material electrochemical properties, descriptions are in the literature, as are the linear polarizations also present only in anodic or cathodic branches of the polarization curves. Anodic branches of the polarization curves were reported, for example, for the characterization of corrosion behaviors of Mg_95_Al_3_Er_2_ and AM60 as cast alloys [58] and AZ91 [72]. Only anodic branches of the polarization curves were used for the description of the corrosion behavior of different dental alloys [73]. On the other hand, only cathodic branches of the polarization curves were compared with the mass loss test results for Mg–8Sn–1Zn–1Al and Mg–8Sn–1Zn–1Al–0.1Mn alloys in [74]. However, no comparison and interpretation of the data obtained using linear polarization, evaluating only anodic or cathodic branches of the curve, are available in the literature according to the authors’ knowledge.

The present paper offers a comparison of the potentiodynamic characteristics of cast AZ91 magnesium alloy obtained by the linear polarization method (from −100 mV to 200 mV vs. E_OCP_), by the cathodic polarization of the specimen (−100 mV vs. E_OCP_), and by the anodic polarization of the specimen (+100 mV vs. E_OCP_). The obtained results are discussed in terms of surface corrosion attack analysis performed by scanning electron microscopy.

## 2. Experimental Material and Methods

### 2.1. Material

A rod of cast AZ91 magnesium alloy was used for the comparison of methods of linear polarization in this study. The chemical composition of the cast AZ91 magnesium alloy was given by the producer, as shown in Table 1.

### 2.2. Microstructure Analysis

To observe the microstructure of the examined AZ91 magnesium alloy, a specimen was cut and embedded into resin. The specimen was ground with 2500 grit SiC paper and polished by diamond paste (1 µm). The polished specimen was etched by picral solution (0.4 g picric acid, 0.3 cm^3^ acetic acid, 0.7 distilled water, and 40 cm^3^ ethanol) for 10 s. The microstructure of the AZ91 was studied by scanning electron microscope (SEM) (ZEISS EVO LS 10, Cambridge, UK) with elemental mapping mode using EDS (OXFORD_INSTRUMENTS_ X-MAX 80 mm^2^, Abingdon, UK).

### 2.3. Electrochemical Measurements

Cast AZ91 magnesium alloy rod was cut into specimens with dimension of Ø15 × 2 mm. Before the test each specimen was ground with 1200 grit SiC paper, cleaned in acetone in an ultrasonic bath (K5 Kraintek, Hradec Králové, Czech Republic) for 5 min to remove residual impurities (grease etc.), rinsed with isopropanol, and dried by warm air. For each measurement five random specimens were prepared.

Electrochemical tests were performed by potentiostat/galvanostat BioLogic VSP-300 in 0.1 M NaCl solution in a three-electrode system: AZ91 alloy was used as the working electrode, a saturated calomel electrode (SCE) as the reference electrode, and a platinum gauze as the counter electrode. All of the measurements were performed at room temperature. Stabilization time of the specimen exposed to the corrosion environment to read the value of open circuit potential (E_OCP_) was 5 min. An area of the specimen exposed to the corrosion environment was 1.0 cm^2^. Measured data were evaluated by BioLogic EC-Lab software (V10.44, BioLogic, Claix, France).

Cathodic polarization curves (CPCs) were measured by polarizing the specimen from the open circuit potential (E_OCP_) to a value of −100 mV vs. E_OCP_. Anodic polarization curves (APCs) were measured by polarizing the specimen surface from E_OCP_ to a value of +100 mV vs. E_OCP_. Potentiodynamic (linear) polarization curves (PCs) containing cathodic and anodic branches together were measured by polarizing the specimen surface to a range from −100 mV to +200 mV vs. E_OCP_, [2,57], to ensure the Tafel region on the anodic branch of the PC. The scan rate was 1 mV·s^−1^. All of the polarization curves were evaluated by Tafel extrapolation (Figure 1) of Tafel regions to obtain i_corr_.

## 3. Results

### 3.1. Microstructural Analysis

**Figure 2:**
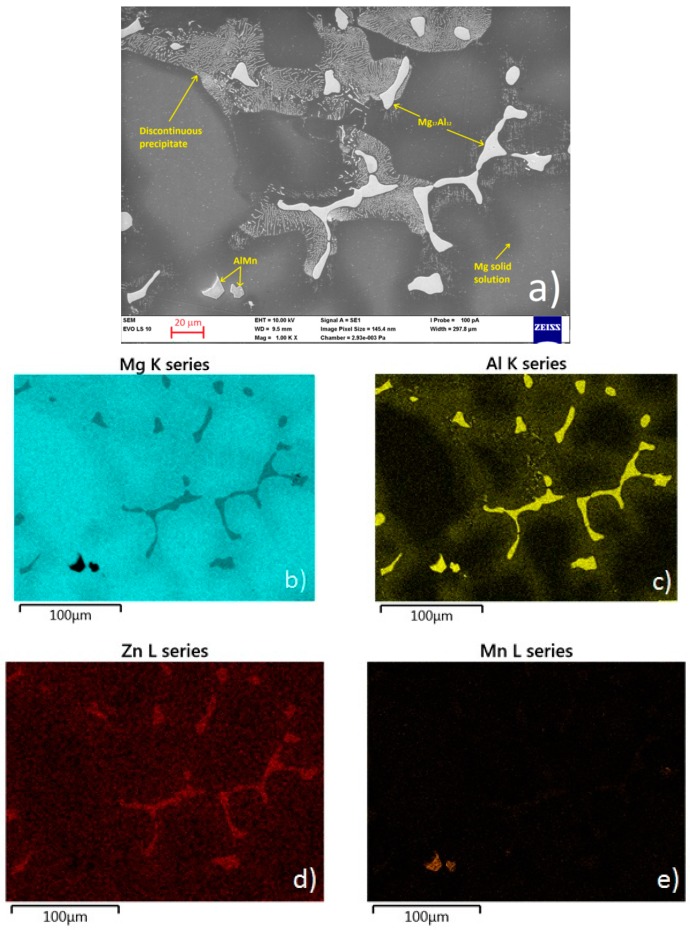
Microstructure of AZ91 magnesium alloy (**a**) and elemental maps: (**b**) magnesium; (**c**) aluminum; (**d**) zinc; and (**e**) manganese (SEM).

The microstructure of cast AZ91 magnesium alloy is shown in Figure 2a. The microstructure of the alloy consists of α-Mg areas (solid solution of alloying elements in magnesium), β phase (Mg_17_Al_12_ intermetallic phase), Mg–Al discontinuous precipitate, and Al–Mn based intermetallic particles.

The distribution of basic alloying elements and present phases was verified by EDS analysis and is presented in a form of elemental maps in Figure 2b–e.

### 3.2. Linear Polarization Measurements

Three different methods of linear polarization were applied to obtain corrosion potential and corrosion current density of the examined cast AZ91 alloy.

**Figure 3:**
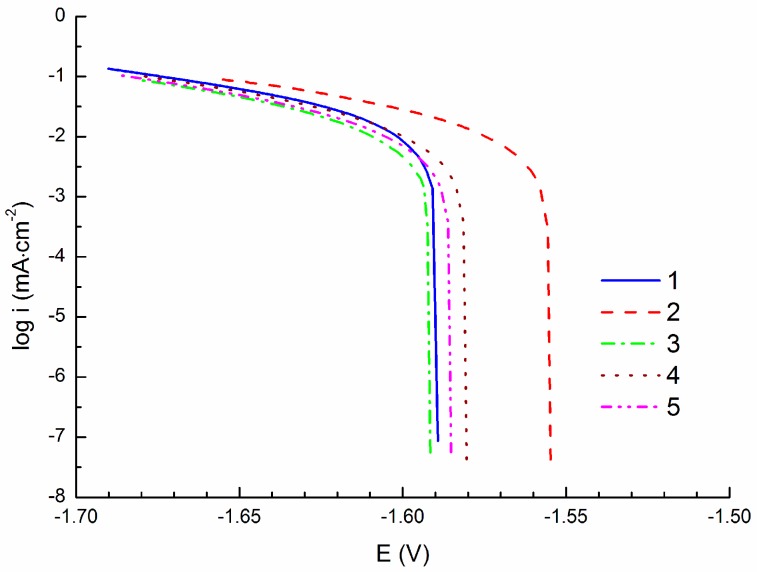
Cathodic polarization curves (CPCs) of AZ91 tested in 0.1 M NaCl solution; −100 mV vs. E_OCP_.

Figure 3 shows CPCs measured by polarization of the specimen to the cathodic region (cathodic polarization; −100 mV vs. E_OCP_). In these conditions hydrogen depolarization occurs on the specimen surface observed via the hydrogen bubble formation. The Tafel region, with a size of approximately 50 mV from the corrosion potential (E_corr_), was used for the data extrapolation, Figure 1, to obtain the corrosion current density i_corr_. The average value of the corrosion current density from the CPCs obtained from five specimens (Table 2) was 15.4 ± 1.0 µA·cm^−2^.

**Figure 4:**
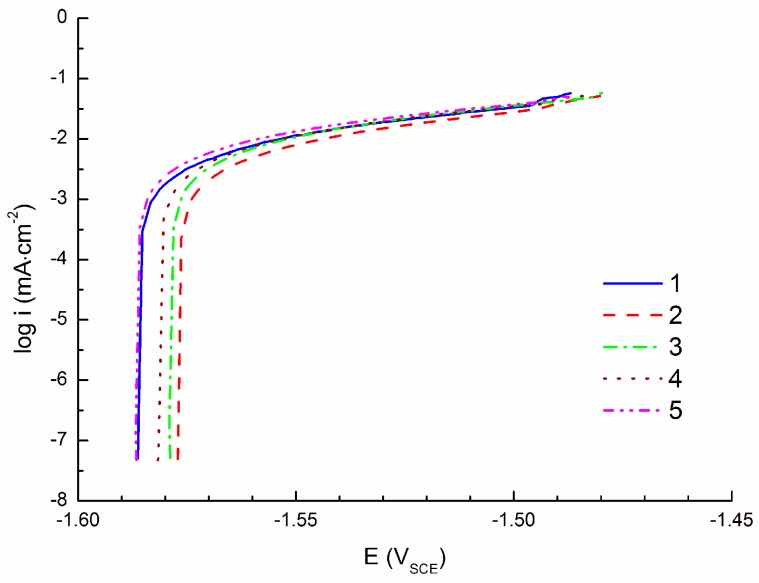
Anodic polarization curves (APCs) of AZ91 tested in 0.1 M NaCl solution; +100 mV vs. E_OCP_.

Measured APCs are shown in Figure 4. The curves were measured by polarization specimen to the anodic region (anodic polarization; +100 mV vs. E_OCP_). In these conditions oxidation (corrosion) of the magnesium alloy occurs. At the end of the curves non-linear regions are observed. This could indicate the beginning of pitting corrosion [75]. With respect to this fact was a potential at the beginning of the non-linear region determined as pitting potential E_pit_. The range of the Tafel region used for the curves’ extrapolation and corrosion current density determination was not of 50 mV (length sufficient for curve extrapolation according to the literature [63,64]), however, the extrapolation was performed on sufficient parts of the curves and the obtained data are given in Table 3. The average value of E_OCP_ determined from APCs was −1.582 ± 0.004 V and the corrosion current density was of 8.0 ± 0.6 µA·cm^−2^.

**Figure 5:**
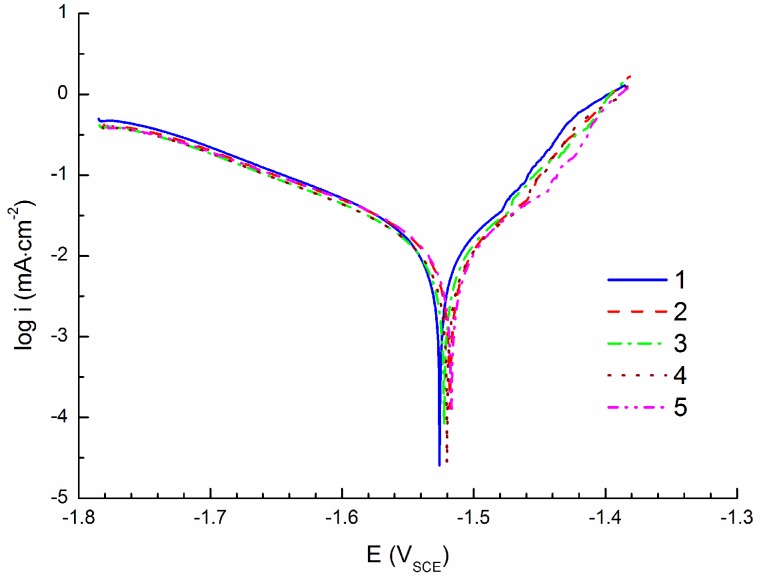
Linear polarization curves (PC) of AZ91 in 0.1 M NaCl solution containing cathodic and anodic parts; from −100 mV to +200 mV vs. E_OCP_.

Obtained linear polarization curves, containing cathodic and anodic polarization branches, commonly used for material electrochemical characterization are shown in Figure 5. Corrosion attack in the form of pitting was observed at the anodic branch of the curves. The attack was characterized by an increase of current density which belongs to the value of pitting potential (E_pit_) [75]. The pitting was observed before the beginning of the Tafel region (between 50 and 100 mV vs. E_OCP_ [63,64]). Therefore, the Tafel region for these anodic branches of the PCs were insufficient to obtain relevant values of i_corr_. On the other hand, the Tafel regions in the cathodic branch of PCs were more than 50 mV vs. E_OCP_. In such situations only the cathodic part of the curve, in combination with E_corr_, for the extrapolation was used. The characteristics obtained from the PCs evaluation are given in Table 4.

The average value of E_OCP_ determined from PCs was −1.575 ± 0.001 V; E_corr_ was of −1.550 ± 0.001 V and the corrosion current density was of 14.7 ± 0.6 µA·cm^−2^.

## 4. Discussion

The microstructure of the cast AZ91 magnesium alloy consists of a solid solution of alloying elements in magnesium in which are randomly distributed AlMn intermetallic phases and intermetallic phases Mg_17_Al_12_ mostly surrounded by areas of Mg–Al discontinuous precipitate (Figure 2). Heterogeneity of the microstructure of AZ91 prepared by casting could have an influence on the scatter of the open circuit potential E_OCP_ values estimated by polarization techniques. Potential characteristics of α-Mg areas and present intermetallic phases are different [76], which is a reason for local corrosion attack of the material on their interface due to the creation of a galvanic cell. 

Values of the open circuit potential E_OCP_ obtained by different polarization methods given in Table 2, Table 3 and Table 4 are very similar for all of the curves. This agreement in measured E_corr_ values indicates only small influence of the microstructure heterogeneity of individual tested specimens. The area exposed to the corrosion environment with the size of 1.0 cm^2^ seems to be sufficient to obtain representative data and to consider the microstructure of the experimental material homogenous. Additionally, no significant differences in the values of the corrosion potential determined by Tafel analysis evaluating curves obtained by different polarization methods were determined. However, differences in the obtained values of the corrosion current density i_corr_ were observed (Table 5). Lower values of i_corr_ determined by ACP (Table 5) suggest the decrease of the corrosion process rate. This could be explained by the corrosion process evolution and creation of the corrosion products on the specimen surface comparing to the specimens polarized to the cathodic region. Corrosion products, with lower electric conductivity when compared to the magnesium alloy itself, act also like a barrier, which protects the basic material against corrosion environment.

On the other hand, corrosion current density i_corr_ obtained by polarization of the specimen to the cathodic area have similar values (Table 5) as the values obtained by the linear polarization measurement. In comparison with anodic polarization measurements, the cathodic polarization measurements do not affect the measured surface. Polarization measurements of the cathodic area do not lead to corrosion (respectively, oxidation) of the material. Only reduction of hydrogen, so called hydrogen depolarization, occurs during cathodic polarization and the material surface does not react with the corrosion environment due to the specimen polarization to more negative values than E_OCP_. The surface of the material does not react with the NaCl solution and the creation of a protecting MgO layer is eliminated.

The value of corrosion current density i_corr_ obtained by CPC and PC are higher than values obtained by APC due to the direct contact of the specimen and the corrosion environment. 

The similarity of the corrosion current densities obtained from PCs and CPCs could also be influenced by the extrapolation of the obtained curves, when mainly cathodic branches of the PCs were used for extrapolation due to the pitting corrosion and subsequently small Tafel region on the anodic branch of the PCs. The range of the Tafel region used for the linear extrapolation of the obtained curves was around 50 mV in the case of CPCs (the exact range from 50 mV from the E_corr_ to the value of about 100 mV from E_corr_). In the case of ACPs the range was from 30–48 mV (the exact range from 50 mV from the E_corr_ to the value of about 80–98 mV from E_corr_). Evaluation of PCs did not follow the theoretical limit according to the literature in the case of extrapolation of the anodic branch of the curve. In the case of the anodic branch the range for the Tafel extrapolation started at 26–40 mV from E_corr_. However, in all of the cases the coefficient of reliability of the line extrapolating the curvilinear part reached the minimum value of 0.95. The determined value of E_OCP_ was considered as E_corr_ in the case of CPS and APC curve evaluations.

**Figure 6:**
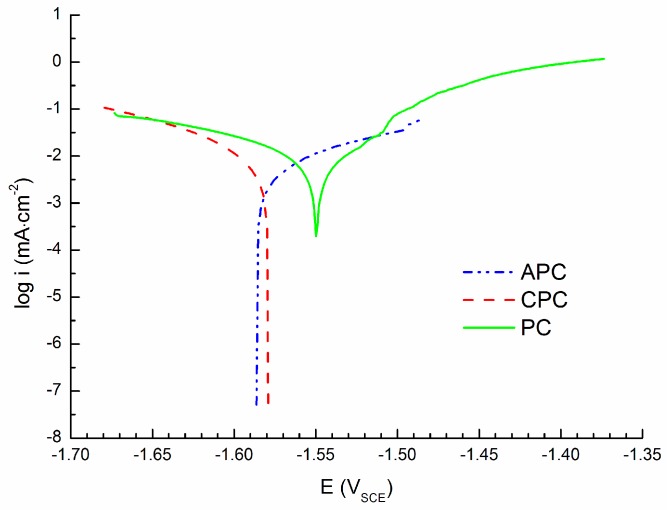
Comparison of polarization techniques of AZ91 in 0.1 M NaCl solution (CPC, APC, PC).

Polarizing the specimen only to the cathodic or only to the anodic region to obtain E_OCP_ can be considered, according to extrapolation shown in Figure 1, as the corrosion potential E_corr_. Using different methods of specimen polarization (CPC, APC, and PC), different values of corrosion potential, E_corr_, were obtained. Corrosion potentials obtained from CPC and APC have almost the same value, while the value of corrosion potential obtained from PC was shifted to more positive values (Figure 6). 

**Figure 7:**
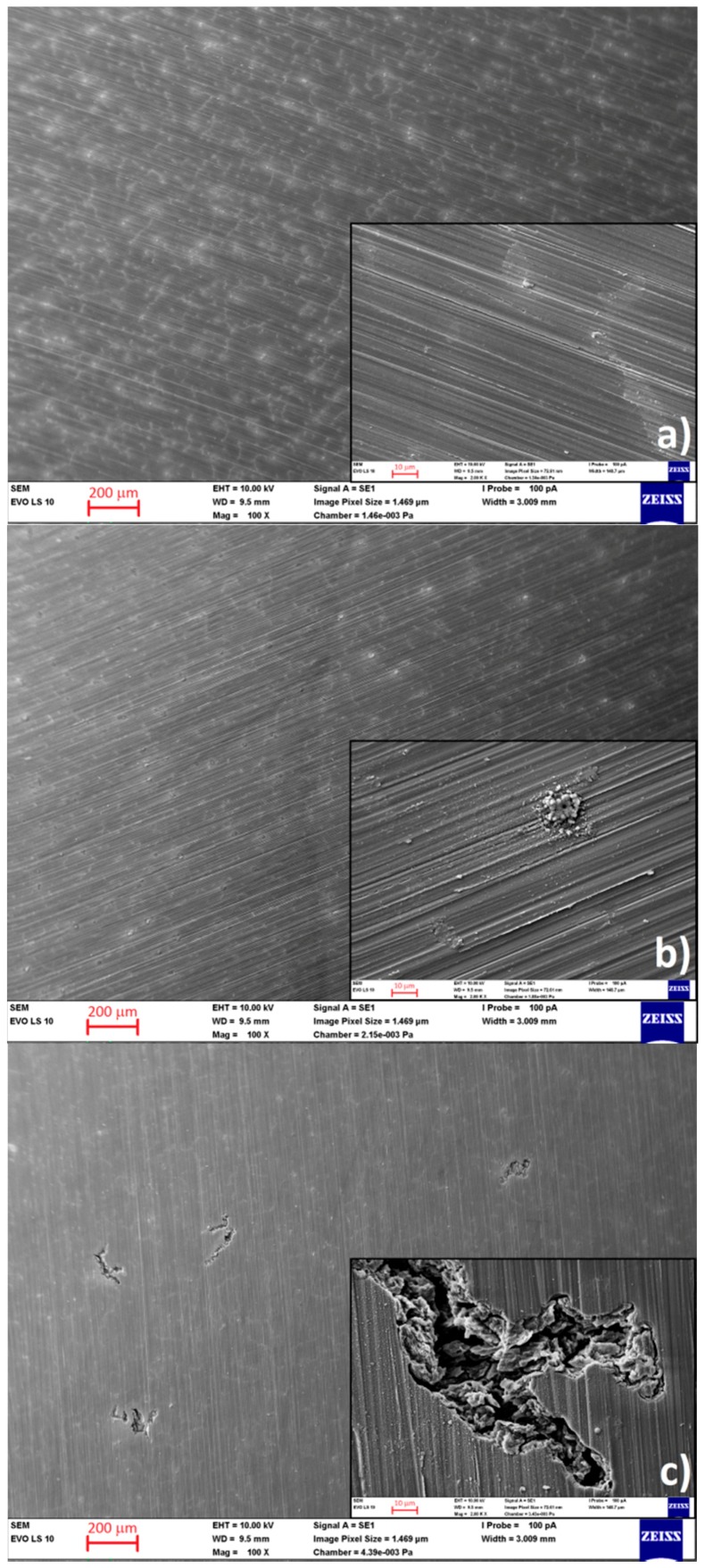
Surface of AZ91 magnesium alloy after electrochemical measurements: (**a**) cathodic polarization; (**b**) anodic polarization; and (**c**) linear polarization.

Figure 7 shows the surface of the AZ91 magnesium alloy after electrochemical measurements. Lighter colored areas on the surface are intermetallic phases and discontinuous precipitate particles present in the microstructure of the alloy (Figure 2). There is no significant corrosion attack observed on the specimen surfaces after cathodic polarization measurements (Figure 7a). Even though a small difference in the determined E_corr_ values were observed in the case of the CPC measured specimens, SEM surface analysis did not reveal significant differences in the corrosion attack of individual specimens. The inspected area with a size of 1 cm^2^ was sufficiently large to allow consideration of the specimen microstructure as homogenous. Specimens measured by anodic polarization (Figure 7b) have corrosion products on their surface. On the specimen surface some localities with higher evolution of corrosion products were present. The areas preferentially attacked by corrosion served as initiation sites for pitting corrosion, which would develop during longer exposition of the material to the 0.1 M NaCl corrosion environment. The localized corrosion products evolution corresponds to the starting pitting corrosion attack observed on APCs. Significant corrosion occurred during linear polarization (PC) measurements (Figure 7c). Areas attacked by pitting corrosion are already well developed and visible on the specimen surface. 

The difference in the corrosion attack of differently polarized specimens can be explained by reactivity of the material. Before each polarization measurement 5 min stabilization was applied. During this period the alloy reacted with the corrosion environment and the specimen surface was affected by formation of the corrosion products. Within the cathodic polarization only hydrogen evolution on the surface was observed. Hydrogen bubbles created on the specimen surface removed corrosion products created on the surface during the stabilization. The specimen surface observed after cathodic polarization did not exhibit any signs of the corrosion attack (Figure 7a). However, in the case of linear polarization, the cleaned specimen surface (with corrosion products removed during the cathodic polarization part) was more reactive during the following anodic polarization part, than the surface of the specimen polarized only to the anodic area, and pitting evolution was observed on the specimen surface (Figure 7c). Additionally, not yet developed pitting was observed on the specimen surface in the form of localized areas of corrosion products, which are comparable with areas observed on the surface of the specimen after anodic polarization. 

Formation of corrosion products on the specimen surface during 5 min of stabilization before polarization can also explain the differences in i_corr_. The lowest value of the i_corr_ in the anodic polarization case could be caused by the oxide layer protecting the specimen surface and slowing the corrosion process of the specimen during the anodic polarization. This protecting layer was, in the case of cathodic polarization and linear polarization (in the cathodic part of the process), removed by hydrogen evolution on the specimen surface.

## 5. Conclusions

This study investigated the effect of using a polarization method on the obtained values of electrochemical characteristics describing the corrosion behavior of cast AZ91 magnesium alloy. The obtained results could be formulated as follows:

1. The microstructure of the examined AZ91 cast magnesium alloy was considered to be homogenous from the macroscopic point of view and homogenous corrosion behavior was observed.
2. Corrosion current density of i_corr_ = 15.4 ± 1.0 µA·cm^−2^ obtained by cathodic polarization is similar to the corrosion current density i_corr_ = 14.7 ± 0.6 µA·cm^−2^ obtained by linear polarization. On the other hand, corrosion current density i_corr_ = 8.0 ± 0.6 µA·cm^−2^ obtained by anodic polarization is significantly lower comparing to the previous methods.
3. Corrosion potentials obtained from PC are shifted to more positive values of potential compared to the ACP and CPC.
4. Significant pitting corrosion was observed only in the case of linear polarization measurements. Localized corrosion product areas were observed on PC and APC specimens.

## Figures and Tables

**Table 1:** Chemical composition of studied cast AZ91 magnesium alloy.

Alloy	Chemical Composition (wt%)								
	Al	Zn	Mn	Si	Fe	Be	Ni	Cu	Mg
AZ91	8.7	0.65	0.25	0.006	0.003	0.0008	0.0006	0.0005	rest

**Table 2:** Electrochemical characteristics of AZ91 in 0.1 M NaCl solution determined by cathodic polarization.

Sample	1	2	3	4	5
E_OCP_ (V)	−1.585	−1.555	−1.585	−1.579	−1.580
Range of Tafel region (mV)	~50	~50	~50	~50	~50
i_corr_ (µA·cm^−2^)	15.0	14.2	16.5	14.5	16.7

**Table 3:** Electrochemical characteristics of AZ91 in 0.1 M NaCl solution determined by anodic polarization.

Sample	1	2	3	4	5
E_OCP_ (V)	−1.586	−1.577	−1.579	−1.582	−1.587
E_pit_ (V)	−1.496	−1.497	−1.481	−1.493	−1.494
Range of Tafel region (mV)	40	30	48	39	43
i_corr_ (µA·cm^−2^)	7.7	7.0	8.6	8.2	8.6

**Table 4:** Electrochemical characteristics of AZ91 tested in 0.1 M NaCl solution measured by linear polarization curves.

Sample	1	2	3	4	5
E_OCP_ (V)	−1.576	−1.577	−1.575	−1.573	−1.574
E_corr_ (V)	−1.551	−1.552	−1.550	−1.550	−1.549
E_pit_ (V)	−1.511	−1.518	−1.524	−1.522	−1.515
Range of ACP Tafel region (mV)	−10	−16	−24	−22	−16
Range of CPC Tafel region (mV)	>50	>50	>50	>50	>50
i_corr_ (µA·cm^−2^)	14.9	13.4	15.1	14.9	15.0

**Table 5:** Comparison of polarization techniques of AZ91 tested in 0.1 M NaCl solution.

Technique	E_OCP_ (V)	E_corr_ (V)	i_corr_ (µA·cm^−2^)
CPC	−1.577 ± 0.011	−1.577 ± 0.011 ^1^	15.4 ± 1.0
APC	−1.582 ± 0.004	−1.582 ± 0.004 ^1^	8.0 ± 0.6
PC	−1.575 ± 0.001	−1.550 ± 0.001	14.7 ± 0.6

^1^ E_corr_ obtained from E_OCP_.
